# Evaluation of simple diagnostic parameters in acute kidney injury in hospitalized patients—diagnostic recommendations for non-nephrologists

**DOI:** 10.1007/s11739-023-03365-x

**Published:** 2023-07-15

**Authors:** Anna Buckenmayer, Nadja Siebler, Christian S. Haas

**Affiliations:** Department of Internal Medicine, Nephrology & Intensive Care Medicine, Phillips University, Baldinger Straße 1, 35043 Marburg, Germany

**Keywords:** Acute kidney injury, Acute tubular necrosis, URINE diagnostic, Urine sodium, Renal failure index

## Abstract

Acute kidney injury (AKI) is very common in hospitalized patients, affecting patient’s mortality and morbidity. Major causes are prerenal AKI and acute tubular necrosis (ATN). Even though a variety of parameters/indices exist, their reliability and practicability are controversial: in fact, there is a need for a simple diagnostic approach for AKI in in-patients with parameters easily obtained in any hospital. The objective of the study was: (1) to assess reliability of simple laboratory parameters/indices to differentiate pre-/intrarenal AKI; (2) to evaluate the most reliable and feasible parameters/indices; and (3) to identify the possible impact of confounding factors. Retrospectively, in-patients with AKI hospitalized in 2020 in a university nephrology department were included. Spot urine and 24-h collection urine was analyzed with urine sodium (U_Na_), urine specific gravity (U_SG_), fractional excretion of sodium (FE_Na_), fractional excretion of urea (FE_Urea_), urine osmolality (U_Osm_), urine to plasma creatinine ratio (U_Cr_/P_Cr_) and renal failure index (RFI). Overall, 431 patients were included. U_Na_, U_Osm_, U_SG_ and RFI showed high specificity > 85% for prerenal AKI, U_Na_ and RFI provided good specificity for ATN. Loop diuretics, ACE inhibitors/AT1 blockers or pre-existing chronic kidney disease had no impact. In patients with AKI, U_Na_, U_SG_ and RFI: (1) proved to be very specific for prerenal AKI and showed high sensitivity for ATN; (2) can be easily determined using serum and spot urine; and (3) are not confounded by medication or comorbidities. These parameters/indices are helpful to identify the aetiology of AKI and to guide therapy, thereby improving patients’ safety and outcome.

**Supplementary Information:**

The online version contains supplementary material available at 10.1007/s11739-023-03365-x.

## Introduction

Acute kidney injury (AKI) is one of the most common diagnoses in hospitalized patients and has relevant impact on morbidity and mortality (1). AKI is defined as a sudden decline in kidney function, classified into three stages, most commonly using the AKIN or—more recently—the KDIGO criteria (2, 3). In hospitalized patients, major causes are acute tubular necrosis (ATN, 45%) and prerenal AKI (21%), whereas other reasons like obstructive nephropathy or glomerular diseases are rare (4). An early and precise diagnosis with respect to the aetiology of AKI is crucial for guiding an adequate therapy, thereby improving patients’ outcome (5). While postrenal causes can be easily excluded using ultrasound, the distinction between pre- and intrarenal AKI is more challenging. Lack of clinical symptoms and competing risk factors in hospitalized patients, such as exposure to contrast agents, nephrotoxic medication, hypotension and infections, makes a diagnostic workup and identifying the underlying cause difficult; this is even more complicated in the simultaneous presence of several potential underlying and/or contributing causes. The recommended diagnostic approach consists of a thorough clinical examination, laboratory blood and urine parameters, urinalysis including a urine sediment and in certain cases obtaining histopathology by a kidney biopsy.

In recent years, new laboratory parameters and biomarkers have been proposed to foster establishing an accurate diagnosis in the case of impaired kidney function, and attempts have been made for using them to predict kidney recovery (6–9). In hospitalized patients, a precise diagnosis is crucial, thereby leading to different therapeutic approaches: prerenal AKI may be primarily addressed with fluid administration in hypovolemic or with modified drug therapy to address congestive heart failure. In ATN, adequate volume management is supportive but identifying and treating the underlying cause is crucial. In fact, inadvertent fluid overload may lead to adverse events, e.g., pulmonary oedema, thereby increasing mortality (10, 11). Of note, due to the often complex aetiology of AKI, early consultation of a nephrologist has been shown to have significant impact on patients’ outcome (12). Nevertheless, many hospitals do not provide nephrology knowledge or services on-site; e.g., in Germany, about 400 nephrologists are hospital-based physicians, of whom more than 90% are working at university and maximum care hospitals (13, 14). Thus, in smaller hospitals of primary or secondary care, patients with advanced renal failure are often seen and treated only by physicians with knowledge of internal medicine or by intensive care specialists. Of note, ATN as an important differential diagnosis to prerenal AKI is not widely known by non-nephrologists, resulting in a probable high percentage of misdiagnosis in AKI patients (15). In addition, use of advanced diagnostic parameters and/or biomarkers for renal damage is rather uncommon in daily clinical practice, most likely due to lack of availability, practicability or costs in a non-nephrological setting. It has been a matter of debate if simple to obtain parameters and indices of renal damage are reliable and useful in clinical practice (16–18).

The objective of the present study was: (1) to assess reliability of simple laboratory parameters and indices to differentiate pre- and intrarenal causes; (2) to evaluate the most reliable and feasible parameters and indices; and (3) to identify the possible impact of confounding factors on the diagnostic workup, such as comorbidities, pre-existing chronic kidney disease and medication. Overall, the study should help to guide therapy in patients with AKI until a nephrological co-assessment can be provided, thereby improving patient safety and outcome.

## Methods

In this retrospective cross-sectional, descriptive study at nephrology ward at the University Hospital in Marburg, Germany, in-patients with an established diagnosis of AKI as indicated in the discharge letter between January 1st, 2020, and December 31st, 2020, were identified. Exclusion criteria were age < 18 years and patients with a functional renal transplant. AKI was defined according to AKIN criteria, using serum creatinine and urine output. At time of presentation, the cause of AKI was suggested by the nephrologist in charge. Patients with obstructive nephropathy as determined by ultrasound were transferred to urological services and excluded. Medical history, clinical signs and symptoms, imaging as well as laboratory parameters were regularly used to assess volume status and to identify the most likely reason for AKI based on routine nephrology care and clinical experience. The complex diagnostical approach included differentiated proteinuria analysis, measurement of urinary concentration of electrolytes, creatinine, and osmolality in spot urine or/and 24 h collection urine, and serum parameters to differentiate acute and chronic components of kidney injury. A final diagnosis of prerenal or intrarenal AKI was established to the nephrologists’ discretion by considering the development of diuresis, laboratory values and the clinical course.

In all patients, data on aetiology of kidney injury, renal disease in case of pre-existing CKD, comorbidities, and on medication, including ACE inhibitors, AT1 blockers and diuretics, were assessed from the patients’ charts. In addition, length of hospital stay, time to readmission within the observational period and multiple serological und urine parameters were gathered. Close attention was paid to urine sodium and the following urinary diagnostic indices as suggested by *Schrier *et al*. *(19): fractional excretion of sodium (FE_Na_), fractional excretion of urea (FE_Urea_), urine osmolality (U_Osm_), urine to plasma creatinine ratio (U_Cr_/P_Cr_) and renal failure index as potential markers and indices to differentiate between pre- and intrarenal causes of AKI (formulas Table 1). For this calculation 24-h collection urine samples were used, when initiated within the first 24 h after admission. Additionally, a differentiated proteinuria diagnostic including albumin and α1-microglobulin was performed.

**Table 1:** Formulas for urinary diagnostic indices

Urine to plasma creatinine (U_Cr_/P_Cr_)	$\frac{{U}_{\mathrm{Cr}}}{{P}_{\mathrm{Cr}}}$	Renal failure index (RFI)	$\frac{{U}_{\mathrm{Na}}\times {P}_{\mathrm{Cr}}}{{U}_{\mathrm{Cr}}}$
Fractional excretion of sodium (FE_Na_)	$\frac{{U}_{\mathrm{Na}}\times {P}_{\mathrm{Cr}}}{{U}_{\mathrm{Cr}}\times {P}_{\mathrm{Na}}}\times 100$	Fractional excretion of urea (Fe_Urea_)	$\frac{{U}_{\mathrm{Urea}}\times {P}_{\mathrm{Cr}}}{{U}_{\mathrm{Cr}}\times {P}_{\mathrm{Urea}}}\times 100$

*U*_*Cr*_ urine creatinine, *P*_*Cr*_ plasma creatinine, *U*_*Na*_ urine sodium, *P*_*Na*_ plasma sodium, *U*_*Urea*_ urine urea, *P*_*Urea*_ plasma urea

Data were analyzed using SPSS statistics (version 28) and Excel (version 16.0). Linear and Phi correlation was performed between urine parameters and the underlying cause of kidney injury, and sensitivity, specificity, positive and negative predictive values (PPV and NPV) as well as positive likelihood ratio were calculated for prerenal AKI and ATN. Data are presented as mean ± standard deviation, unless otherwise stated; where applicable, the confidence interval (CI) is shown. A *p* value < 0.05 was considered statistically significant. The study was given a waiver by the Ethics Committee, Philipps University, Marburg, Germany (11/2021 RS 21/88).

## Results

A total of 431 patients were included in the study. Of those, 118 patients were transferred from other departments in the hospital (27.4%), about a quarter of patients included in the study were treated on the ICU at some point. Mean age was 71.6 ± 14.6 years at time of admission with a male predominance (63.3%). Baseline characteristics of all patients are shown in Table 2. Nearly half the study population presented with severe AKI (*n = *210, 48.7%). Prerenal causes were the most common reasons for renal failure (*n = *309, 71.7%), while ATN was the predominant reason for loss of kidney function in intrarenal AKI (*n = *64, ATN *n = *47, 73.4%). In 41 cases (9.5%), a combination of multiple reasons was present, mainly a combination of ATN with a pre- or postrenal component. At date of admission, 40.2% of patients had pre-existing CKD, predominantly in advanced stages.

**Table 2:** Baseline characteristics by Age Group. AKI, acute kidney injury, CKD chronic kidney disease

Age [years]	18–39 (*n = *22)	40–49 (*n = *11)	50–59 (*n = *51)	60–69 (*n = *86)	≥ 70 (*n = *261)	all (*n = *431)
Male sex	14 (63.6%)	7 (63.6%)	36 (70.5%)	65 (75.6%)	151 (57.9%)	**273 (63.3%)**
Stage of AKI						
AKIN 1	6 (27.3%)	1 (9.1%)	10 (19.6%)	23 (26.7%)	80 (30.7%)	**120 (27.8%)**
AKIN 2	6 (27.3%)	0 (0.0%)	10 (19.6%)	20 (23.3%)	65 (24.9%)	**101 (23.4%)**
AKIN 3	10 (45.5%)	10 (90.9%)	31 (60.8%)	43 (50.0%)	116 (44.4%)	**210 (48.7%)**
Aetiology of AKI						
Prerenal	14 (63.6%)	7 (63.6%)	34 (66.7%)	58(67.4%)	196 (75.1%)	**309 (71.7%)**
Intrarenal	8 (36.4%)	3(27.3%)	8 (15.7%)	16 (18.6%)	29 (11.1%)	**64 (14.8%)**
Postrenal	0 (0.0%)	0 (0.0%)	5 (9.8%)	1 (1.2%)	11 (4.2%)	**17 (3.9%)**
Combined	0 (0.0%)	1 (9.1%)	4 (7.8%)	11 (12.8%)	25 (9.6%)	**41 (9.5%)**
Pre-existing CKD	3 (13.6%)	2 (18.2%)	21 (41.2%)	36 (42.4%)	148 (58.0%)	**210 (49.5%)**
G1	0 (0.0%)	0 (0.0%)	2 (3.9%)	1 (1.2%)	0 (0%)	**3 (0.7%)**
G2	0 (0%)	0 (0%)	3 (5.9%)	3 (3.5%)	7 (2.7%)	**13 (3.1%)**
G3a	1 (4.5%)	0 (0.0%)	5 (9.8%)	7 (8.2%)	18 (7.1%)	**31 (7.3%)**
G3b	1 (4.5%)	0 (0.0%)	6 (11.8%)	8 (9.4%)	41 (16.1%)	**56 (13.2%)**
G4	0 (0.0%)	1 (9.1%)	2 (3.9%)	8 (9.4%)	50 (19.2%)	**61 (14.4%)**
G5	1(4.5%)	1(9.1%)	3 (5.9%)	9 (10.6%)	32 (12.3%)	**46 (10.8%)**

Bold values emphasise the most important results

*AKI* acute kidney injury, *CKD* chronic kidney disease

During hospitalization, dialysis as renal replacement therapy (RRT) was performed in 109 patients (25.2%; continuous RRT, *n = *74; intermittent RRT, *n = *33; peritoneal dialysis, *n = *2); more than half of those (62 patients = 56.8%) required permanent RRT at time of discharge (14.4% of all patients with AKI). Seventy-eight patients (18%) deceased, while six patients (1.4%) were discharged to a palliative setting. At discharge, 191 patients (44.3%) still had impaired renal function with elevated creatinine levels, meeting criteria of acute kidney disease (AKD), which is defined as a persistent reduction of kidney function between 7 and 90 days following AKI ((20), Table 3). In 55.7% of patients, kidney function resolved in less than 7 days.

**Table 3:** Patients’ outcome with respect to kidney function and mortality

Age [years]	18–39 (*n = *22)	40–49 (*n = *11)	50–59 (*n = *51)	60–69 (*n = *86)	≥ 70 (*n = *261)	all (*n = *431)
Discharge with AKD	13 (59.0%)	6 (54.6%)	21 (41.1%)	34 (39.4%)	117 (44.8%)	**191 (44.3%)**
Temporary dialysis	5 (22.7%)	6 (54.5%)	16 (31.4%)	27 (31.4%)	55 (21.1%)	**109 (25.2%)**
Discharge with RRT	1 (4.5%)	3 (27.2%)	6 (11.8%)	10 (11.6%)	42 16.1%)	**62 (14.4%)**
Discharge with palliative care	0 (0.0%)	0 (0.0%)	0 (0.0%)	0 (0.0%)	6 (2.3%)	**6 (1.4%)**
Death	1 (4.5%)	1 (9.0%)	9 (17.6%)	16 (18.6%)	51 (19.5%)	**78 (18.0%)**

Bold values emphasise the most important results

*AKD* acute kidney disease, *RRT* renal replacement therapy

Within the first 24 h of admission, a total of 331 patients (76.8%) received an extended blood and urine diagnostic workup, including collective urine over 24 h and cystatin-C as an alternative marker for glomerular filtration with advantages in the creatinine-blind range. In 87 patients, diagnostic workup was delayed; in 13 patients, collection of 24-h urine wasn’t feasible due to incontinence and refusal of a Foley catheter. In those cases, measurement of electrolytes and determination of proteinuria was performed using spot urine. In the remaining patients, the diagnostic workup was delayed, mainly due to clinical focus on non-renal diseases.

As expected, values of creatinine und cystatine-C showed good correlation (*r = *0.661, *p* < 0.001). At time of admission, 114 patients (34.4%) showed a FE_Na_ below 1%, matching the nephrologists’ diagnosis of prerenal AKI in 83.3% cases (*n = *95). In fact, there was a significant correlation of FE_Na_ < 1% and prerenal AKI (*r = *0.17, *p* < 0.001). Reliability of FE_Na_ was assessed by logistic regression analysis, revealing a sensitivity of 91.4% and specificity of 36.1% for FE_Na_ to predict prerenal AKI. Assessment of urinary sodium concentration was used to help in differentiating between pre- and intrarenal causes, showing a significant correlation of U_Na_ < 30 mmol/l with prerenal AKI (*r = *0.167, *p = *0.002) but not with intrarenal AKI (*r = *0.071, *p = *0.197), when U_Na_ ≥ 30 mmol/l was used. Of note, FE_Na_ > 1% was not correlated with intrarenal AKI (*n = *331, *r = *0.073, *p = *0.181); similarly, fractional excretion of urea (FE_Urea_) < 35% was not helpful to suggest pre- or intrarenal cause of AKI. In fact, FE_Urea_ showed the lowest sensitivity and specificity of all parameters. Of note, specificity of the renal failure index (RFI) < 1 was surprisingly high (85.7%) for prerenal AKI, while an RFI > 1 was very sensitive for an intrarenal cause (80.8%, Table 4).

**Table 4:** Sensitivity and Specificity of urinary diagnostic values and indices(34)

	Prerenal						Intrarenal					
	Cut off	Sensitivity (%)	Specificity (%)	PPV (%)	NPV (%)	LR +	Cut off	Sensitivity (%)	Specificity (%)	PPV (%)	NPV (%)	LR +
Urine specific gravity (U_SG_)	> 1.020	18.8	**88.8**	81.3	29.8	1.7	< 1.012	69.5	56.2	22.4	91	1.6
Urine sodium (U_Na_)	< 30 mmol/l	22.1	**92.3**	88.3	31	2.9	≥ 30 mmol/l	**88.5**	19.0	17	89.8	1.1
Urine osmolality (U_OSM_)	> 500mosm	12.7	**92.1**	80.6	29.2	1.6	< 300mosm	11.8	84.6	12.8	83.3	0.8
Urine to plasma creatinine (U_Cr_/P_Cr_)	> 40	34.7	81.7	83.2	32.5	1.9	< 20	51.8	59.6	19.7	87	1.28
Renal failure index (RFI)	< 1	32.1	**85.7**	85.6	32.4	2.2	> 1	**80.8**	28.7	17.4	88.9	1.1
Fractional excretion of sodium (FE_Na_)	< 1%	39.6	79.1	83.3	33.2	1.9	> 1	73.1	36.6	17.7	87.9	1.2
Fractional excretion of urea (FE_Urea_)	< 35%	54.0	59.8	77.7	33.3	1.3	> 35	60.4	52.2	19.4	87.3	1.3

Bold values emphasise the most important results

*PPV* positive predictive value, *NPV* negative predictive value, *LR* + positive likelihood ratio

Screening for proteinuria via spot urine was positive in 252 cases (58.5%), half of these patients presented with pre-existing glomerular damage and CKD (*n = *129, 51.2%). While specific gravity in spot urine was helpful to identify intrarenal disease, this parameter showed no correlation with urine osmolality in 24-h urine (*r = − *0.002, *p = *0.975, *n = *289). Regarding the extended proteinuria diagnostic in 24-h urine, an increased concentration of α1-microglobuline in 24-h urine was indicative for ATN with remarkable sensitivity (92%).

To identify potential limitations of these parameters and indices, co-morbidities and co-medication and their impact on the studied parameters and indices were evaluated: Of interest, the intake of loop diuretics did not significantly affect sensitivity and specificity of U_Na_, U_SG_ and RFI. A similar observation was made when ACE inhibitors or AT1 blockers were used (Table 5). Comorbidities (hypertension, diabetes mellitus, chronic heart failure, urinary tract infection) as well didn´t have an impact on parameters (Supplementary Material 1).

**Table 5:** Sensitivity and Specificity of urinary diagnostic values and indices in patients taking comedication

	Prerenal						Intrarenal					
	Cut off	Sensitivity (%)	Specificity (%)	PPV (%)	NPV (%)	LR +	Cut off	Sensitivity (%)	Specificity (%)	PPV (%)	NPV (%)	LR +
Patients taking loop diuretics (*n* = 182)												
Urine specific gravity (U_SG_)	> 1.020	12.2	**95.7**	87.5	30.8	2.8	< 1.012	76.0	36.5	18	89.3	1.2
Urine sodium (U_Na_)	< 30 mmol/l	20.0	**92.3**	87.5	30	2.6	> 30 mmol/l	**90.9**	18.0	16.7	91.7	1.1
Urine osmolality (U_OSM_)	> 500mosm	13.7	**94.6**	87.5	28.5	2.5	< 300mosm	9.5	83.9	9.5	83.9	0.6
Urine to plasma creatinine (U_Cr_/P_Cr_)	> 40	26.7	89.7	87.5	31.3	2.6	< 20	59.1	55.7	19.4	88.3	1.3
Renal failure index (RFI)	< 1	28.6	**92.3**	90.9	32.4	3.7	> 1	**86.4**	24.6	17.1	90.9	1.1
Fractional excretion of sodium (FE_Na_)	< 1%	31.4	82.1	82.5	30.8	1.8	> 1%	72.7	28.7	15.5	85.4	1
Fractional excretion of urea (Fe_Urea_)	< 35%	55.2	56.4	77.3	31.9	1.3	> 35%	63.6	54.9	20.3	89.3	1.4
Patients taking ACE inhibitors or AT1 blockers (*n* = 215)												
Urine specific gravity (U_SG_)	> 1.020	11.5	**86.2**	65.2	30.3	0.8	< 1.012	66.7	46.2	19	88	1.2
Urine sodium (U_Na_)	< 30 mmol/l	17.6	**89.1**	78.6	32.2	1.6	≥ 30 mmol/l	**83.9**	14.8	17	81.5	1
Urine osmolality (U_OSM_)	> 500mosm	13.7	**90.6**	72.2	30.8	1.5	< 300mosm	6.7	82.6	7.4	81	0.4
Urine to plasma creatinine (U_Cr_/P_Cr_)	> 40	30.4	80.0	77.6	33.6	1.5	< 20	45.2	56.4	17.7	83.2	1
Renal failure index (RFI)	< 1	29.6	**83.6**	80.4	34.3	1.8	> 1	**77.4**	26.2	18	84.8	1
Fractional excretion of sodium (FE_Na_)	< 1%	33.6	76.4	76.4	33.6	1.4	> 1%	67.7	31.5	17	82.5	1
Fractional excretion of urea (Fe_Urea_)	< 35%	54.8	50.9	71.6	33.3	1.1	> 35%	51.6	54.1	19	84.2	1.1

Bold values emphasise the most important results

*PPV* positive predictive value, *NPV* negative predictive value, *LR* + positive likelihood ratio

## Discussion

Patients with AKI and/or CKD are in imperative need of specific diagnostic steps and adapted care to improve clinical outcome, and consultation of a nephrologist may significantly reduce morbidity and mortality (12, 21, 22). Intrahospital acquired AKI is very common in all medical specialties and should require specific attention due to its relevant impact on patients outcome (1). However, reality in many hospitals may differ significantly since consultation of a nephrologist often does not happen (12, 23) or is delayed—either because of no availability of nephrological services or a lack of physicians’ attention to the relevance of AKI (12). In daily clinical practice, differentiation between prerenal AKI and ATN is challenging, while other causes of intrarenal AKI, like glomerulonephritis or vasculitis are rather rare (~ 4% of all AKI, (4). Especially in non-internal departments, awareness for relevance of acute loss of kidney function is low (24, 25). While correct intrahospital evaluation and subsequent outpatient follow-up by a nephrologist should be the goal in all patients with AKI, this might exceed capacities of most inpatient and outpatient facilities. Additionally, Silver et al. showed in the FUSION trial that many patients decline enrolment in a nephrology care hospital discharge due to hospitalization-related fatigue, reluctance to add more doctors to their health care team or even long travel times (26). Thus, basic knowledge of potential causes as well as of diagnostic and therapeutic measures for AKI in hospitalized patients should be present in all medical disciplines. While a complex and lavish differential diagnostic pathway is often not helpful, a simple, repeatable and cheap basis approach should be preferred.

In the pre-biomarkers´ era for AKI, several less expensive and almost anywhere available urine and serum parameters had been proposed to differentiate between prerenal AKI and ATN, like urinalysis, urine sodium and osmolality, FE_Na_, FE_urea_, U_Cr_/P_Cr_ and RFI. However, data are sparse on the use and reliability in daily clinical practice. The objective of the present study was to evaluate sensitivity and specificity of those parameters and to address their potential limitations with real-life data. Urinalysis of spot urine is the quickest, most commonly used and easiest urine test method in any medical department. The present study showed that specific gravity of the urine—as determined with a dip stick—can give valuable information on the potential aetiology of AKI. Similarly, urine sodium concentration provides reliable information on aetiology regarding pre- and intrarenal differentiation. FE_Na_ has been promoted to be superior since it represents urine sodium excretion in proportion to kidney function (9); an FE_Na_ value of less than 1% is considered to indicate prerenal cause of AKI, due to maximum fluid sodium retention of the kidney to conserve fluid (27), while a higher FE_Na_ rather points to tubular damage. However, the present data did not support the idea of superiority, contradicting some pre-existing data (6, 9, 28). In addition, FE_Na_ is known only by few health care practitioners or is often even determined using 24-h urine, making FE_Na_ a rather impractical tool. Fractional urea excretion (FE_Urea_) and urine osmolality are also often promoted as helpful means (29). However, in the present study, FE_Urea_ showed similar results without any additional beneficial information. Although, 24-h urine is said to be required for calculation of the indices above, this approach is somewhat laborious and not infrequently a barrier for patients, nurses and physicians. To simplify the approach, measurement of sodium in spot urine seems to be a quick and cheap alternative, showing comparable results as demonstrated elsewhere (18, 30, 31). Interestingly, RFI seems to be a little known but helpful index that is easy to obtain in any hospital. In contrast to previous data, certain co-comorbidities and co-medications did not have a significant impact on the examined parameters and indices (32, 33). This is important since drugs targeting the renin–angiotensin–aldosterone-system are very common, making U_Na_, U_SG_ and RFI useful means to differentiate prerenal AKI and ATN in hospitalized patients.

Additional information can be acquired with a simple urine stix to determine if relevant albuminuria or haemoglobinuria are present, potentially hinting at other intrarenal causes of AKI: albumin in the urine is suggestive for glomerular damage, while haemoglobinuria may also point to glomerulopathies or a postrenal cause. In this case, consultation of a specialist should be initiated.

There are some limitations to the study: (1) data collection was performed in a nephrology department with a potential higher prevalence of patients with pre-existing renal diseases, thereby not reflecting in-patients of other departments. On the other hand, in the present setting, a comprehensive serological and urine diagnostic was guaranteed, allowing ideal conditions for evaluating renal indices and parameters and their reliability under different circumstances; (2) the retrospective study design per se has limitations, since data are incomplete, e.g., due to missing follow-up; and (3) although the cause of AKI was established according to the nephrologists’ judgment, there was no second evaluation.

In conclusion, we showed that in hospital-acquired AKI: (1) many indices to differentiate are available, but U_Na_, U_SG_ and RFI have the highest specificity to identify prerenal AKI and the best sensitivity for intrarenal AKI; (2) loop diuretics, ACE-inhibitors and AT1- blockers seem to have no significant impact on these parameters/indices; and (3) U_Na_, U_SG_ and RFI can be measured easily, quick and cost saving using spot urine and serum. These simple tools provide a practicable and quick diagnostic approach for non-nephrologists to differentiate pre- and intrarenal AKI, thereby allowing an early specific therapy and subsequently improving patients’ safety and outcome. However, further prospective studies are needed to evaluate the reliability of this recommendations in clinical practice as a proof of concept.

## Supplementary Information

Below is the link to the electronic supplementary material.

Supplementary file1 (DOCX 43 KB)

## Data Availability

The datasets generated and analyzed during the current study are available from the corresponding author on reasonable request.
