# Supplementary material for: Evaluation of simple diagnostic parameters in acute kidney injury in hospitalized patients—diagnostic recommendations for non-nephrologists
Source: Intern Emerg Med. 2023 Jul 15;18(6):1769–76. doi: 10.1007/s11739-023-03365-x (PMC10504189; doi:10.1007/s11739-023-03365-x)
Supplement: Supplementary file 1 — Supplementary file1 (DOCX 43 KB) [file 11739_2023_3365_MOESM1_ESM.docx]

**Supplementary material:** flowchart of patient selection process

Patients treated on the nephrology ward
**n=1402**

Patients included in study
**n=431**

Patients without AKI
**n=862**

Transplant recipients
**n=109**

**Supplementary material:** Reliability of urinary indices taking into account comorbidities/coinfections.

1. Patients with Diabetes mellitus (n=149)

|  | Prerenal | | | | | | Intrarenal | | | | | |
| --- | --- | --- | --- | --- | --- | --- | --- | --- | --- | --- | --- | --- |
|  | **Cut off** | **Sensitivity** | **Specificity** | **PPV** | **NPV** | **LR+** | **Cut off** | **Sensitivity** | **Specificity** | **PPV** | **NPV** | **LR+** |
| Urine specific gravity (U_SG_) | > 1.020 | 13.3% | 91.4% | 81.3% | 27.4% | 1.5 | < 1.012 | 50% | 53.9% | 14.5% | 87.3% | 1.1 |
| Urine sodium (U_Na_) | <30mmol/l | 20.7% | 90.9% | 85.7% | 30.3% | 2.3 | >30mmol/l | 88.9% | 17.6% | 16% | 90% | 1.1 |
| Urine osmolality (U_OSM_) | >500mosm | 7.5% | 90.6% | 66.6% | 28.2% | **0.8** | <300mosm | 0% | 85.1% | 0% | 81.6% | **0** |
| Urine to plasma creatinine (U_Cr_/P_Cr_) | >40 | 24.1% | 78.8% | 75% | 28.3% | 1.1 | <20 | 50% | 52% | 15.5% | 85.5% | **1** |
| Renal failure index (RFI) | <1 | 27.6% | 84.8% | 82.8% | 30.8% | 1.8 | >1 | 83.3% | 25.5% | 16.5% | 89.7% | 1.1 |
| Fractional excretion of sodium (FE_Na_) | <1% | 33.3% | 78.8% | 80.6% | 31% | 1.6 | >1% | 66.7% | 31.4% | 14.6% | 84.2% | **1** |
| Fractional excretion of urea (Fe_Urea_) | <35% | 50.6% | 39.4% | 68.8% | 23.2% | **0.8** | >35% | 33.3% | 51% | 10.7% | 81.3% | **0.7** |

PPV, positive predictive value; NPV, negative predictive value; LR+, positive likelihood ratio

1. Patients with hypertension (n=299)

|  | Prerenal | | | | | | Intrarenal | | | | | |
| --- | --- | --- | --- | --- | --- | --- | --- | --- | --- | --- | --- | --- |
|  | **Cut off** | **Sensitivity** | **Specificity** | **PPV** | **NPV** | **LR+** | **Cut off** | **Sensitivity** | **Specificity** | **PPV** | **NPV** | **LR+** |
| Urine specific gravity (U_SG_) | > 1.020 | 15.9% | 88.5% | 77% | 30.3% | 1.4 | < 1.012 | 71.8% | 52.2% | 20.4% | 91.5% | 1.5 |
| Urine sodium (U_Na_) | <30mmol/l | 20.4% | 94% | 89.1% | 32.8% | 3.4 | >30mmol/l | 91.7% | 17.1% | 17.1% | 91.7% | 1.1 |
| Urine osmolality (U_OSM_) | >500mosm | 12.9% | 90.8% | 76.9% | 30.4% | 1.4 | <300mosm | 8.6% | 84.3% | 9.4% | 83% | 0.5 |
| Urine to plasma creatinine (U_Cr_/P_Cr_) | >40 | 33.7% | 84.1% | 83.3% | 35% | 2.1 | <20 | 54.1% | 56.9% | 19.2% | 86.7% | 1.3 |
| Renal failure index (RFI) | <1 | 32.1% | 89.6% | 88.1% | 35.3% | 3.1 | >1 | 86.1% | 28% | 18.2% | 91.5% | 1.2 |
| Fractional excretion of sodium (FE_Na_) | <1% | 37.7% | 85.1% | 86% | 36.1% | 2.5 | >1% | 80.6% | 33.7% | 18.5% | 90.3% | 1.2 |
| Fractional excretion of urea (Fe_Urea_) | <35% | 57.4% | 52.9% | 74.4% | 34.3% | 1.2 | >35% | 54.1% | 56% | 19% | 86.4% | 1.2 |

PPV, positive predictive value; NPV, negative predictive value; LR+, positive likelihood ratio

1. Patients with chronic heart failure (n=211)

|  | Prerenal | | | | | | Intrarenal | | | | | |
| --- | --- | --- | --- | --- | --- | --- | --- | --- | --- | --- | --- | --- |
|  | **Cut off** | **Sensitivity** | **Specificity** | **PPV** | **NPV** | **LR+** | **Cut off** | **Sensitivity** | **Specificity** | **PPV** | **NPV** | **LR+** |
| Urine specific gravity (U_SG_) | > 1.020 | 13% | 92.7% | 86.4% | 23% | 1.8 | < 1.012 | 66.7% | 46.2% | 11.7% | 92.9% | 1.2 |
| Urine sodium (U_Na_) | <30mmol/l | 22.7% | 88.2% | 88.2% | 22.7% | 1.9 | >30mmol/l | 80% | 20.5% | 9.1% | 91.2% | 1 |
| Urine osmolality (U_OSM_) | >500mosm | 10.2% | 90.6% | 81.3% | 20.1% | 1.1 | <300mosm | 7.1% | 84.9% | 4.3% | 90.5% | 0.5 |
| Urine to plasma creatinine (U_Cr_/P_Cr_) | >40 | 32.3% | 79.4% | 86% | 23.1% | 1.6 | <20 | 46.7% | 61.2% | 10.6% | 92.1% | 1.2 |
| Renal failure index (RFI) | <1 | 34.1% | 85.3% | 90% | 25% | 2.3 | >1 | 66.7% | 29.8% | 8.6% | 90% | 1 |
| Fractional excretion of sodium (FE_Na_) | <1% | 37.9% | 79.4% | 87.7% | 24.8% | 1.8 | >1% | 60% | 33.8% | 8.3% | 89.5% | 0.9 |
| Fractional excretion of urea (Fe_Urea_) | <35% | 58.8% | 44.1% | 80.2% | 21.7% | 1.1 | >35% | 40% | 58% | 8.7% | 90.6% | 1 |

PPV, positive predictive value; NPV, negative predictive value; LR+, positive likelihood ratio

1. Patients with urinary tract infection (n=133)

|  | Prerenal | | | | | | Intrarenal | | | | | |
| --- | --- | --- | --- | --- | --- | --- | --- | --- | --- | --- | --- | --- |
|  | **Cut off** | **Sensitivity** | **Specificity** | **PPV** | **NPV** | **LR+** | **Cut off** | **Sensitivity** | **Specificity** | **PPV** | **NPV** | **LR+** |
| Urine specific gravity (U_SG_) | > 1.020 | 9.7% | 84.2% | 70% | 19.8% | 0.6 | < 1.012 | 57.1% | 47.6% | 8.3% | 93% | 1.1 |
| Urine sodium (U_Na_) | <30mmol/l | 18% | 100% | 100% | 26.5% | 0 | >30mmol/l | 100% | 15.3% | 10.3% | 100% | 1.2 |
| Urine osmolality (U_OSM_) | >500mosm | 11.9% | 100% | 100% | 24.6% | 0 | <300mosm | 0% | 78.6% | 0% | 90.2% | 0 |
| Urine to plasma creatinine (U_Cr_/P_Cr_) | >40 | 30.6% | 73.7% | 79.2% | 24.6% | 1.2 | <20 | 28.6% | 52.7% | 5.4% | 88.6% | 0.6 |
| Renal failure index (RFI) | <1 | 23% | 83.3% | 82.4% | 24.2% | 1.4 | >1 | 71.4% | 20.8% | 8.1% | 88.2% | 0.9 |
| Fractional excretion of sodium (FE_Na_) | <1% | 24.6% | 72.2% | 75% | 22% | 0.9 | >1% | 57.1 % | 26.4% | 7% | 86.3% | 0.8 |
| Fractional excretion of urea (Fe_Urea_) | <35% | 55.7% | 44.4% | 77.3% | 22.9% | 1 | >35% | 57.1% | 56.9% | 11.4% | 93.2% | 1.3 |

PPV, positive predictive value; NPV, negative predictive value; LR+, positive likelihood ratio
